# Association between pregravid liver enzyme levels and gestational diabetes in twin pregnancies: a secondary analysis of national cohort study

**DOI:** 10.1038/s41598-021-98180-9

**Published:** 2021-09-21

**Authors:** Jae-Young Park, Woo Jeng Kim, Yoo Hyun Chung, Bongseong Kim, Yonggyu Park, In Yang Park, Hyun Sun Ko

**Affiliations:** 1 Department of Obstetrics and Gynecology, Seoul St. Mary’s Hospital, College of Medicine, The Catholic University of Korea, 222, Banpo-daero, Seocho-gu, Seoul, 06591 Republic of Korea; 2 Department of Obstetrics and Gynecology, Daejeon St. Mary’s Hospital, College of Medicine, The Catholic University of Korea, Seoul, Republic of Korea; 3 Department of Biostatistics, College of Medicine, The Catholic University of Korea, Seoul, Republic of Korea

**Keywords:** Medical research, Risk factors

## Abstract

Multiple pregnancies are prone to gestational diabetes mellitus (GDM). This study investigated the association between pregravid liver enzyme levels and the development of GDM in a twin pregnancy. Women who had the National Health Screening Examination and delivered their twin babies within one year were enrolled. Pregravid liver enzyme levels were divided into high and low level. Risks for developing GDM by high levels of liver enzymes were analyzed, in subgroups by pregravid obesity or metabolic syndrome. Among the 4348 twin pregnancies, 369 women (8.5%) developed GDM not requiring insulin treatment (GDM − IT), and 119 women (2.7%) developed GDM requiring insulin treatment(GDM + IT). High levels of pregravid GGT and ALT were related to risks of GDM + IT not only in women with obesity or metabolic syndrome (odds ratio[OR] 6.348, 95% confidence interval [CI] 2.579–15.624 and OR 6.879, 95% CI 2.232–21.204, respectively), but also in women without obesity (OR 3.05, 95% CI 1.565–5.946) or without metabolic syndrome (OR 3.338, 95% CI 1.86–5.992), compared to in women with low levels of those. However, there were no significant associations in the pregravid ALT and GGT levels and risks for development of GDM − IT, unrelated to pregravid obesity or metabolic syndrome. Therefore, this study suggests that women with high levels of pregravid GGT and ALT need to recognize their increased risk of GDM + IT, regardless of pregravid obesity or MetS, when they get pregnant twin.

## Introduction

Gestational diabetes mellitus (GDM) is a common obstetric complication, and its prevalence has been increasing worldwide^1^. Risk factors for GDM include advanced maternal age, pregravid obesity or metabolic disease, family history of diabetes, parity, multiple pregnancy, assisted reproduction technology (ART) treatment, and the specific races of Black African and South Asian^2–4^. GDM is associated with adverse obstetric outcomes such as large for gestational age (LGA) neonates, shoulder dystocia, neonatal respiratory morbidity, and cesarean delivery^5^, also with a long-term risk of type 2 DM (T2DM)^6^. Moreover, GDM requiring insulin treatment (GDM + IT) compared with GDM not requiring insulin treatment (GDM − IT), and Asian origin compared with Caucasian, were suggested as predictive factors for the development of T2DM, among women with GDM^7^. Therefore, Asian women with GDM + IT can be considered to be a very vulnerable risk group for T2DM**.** In Republic of Korea, the prevalence of GDM increased abruptly, from 3.86% in 2007 to 9.5% in 2010^8^.

Advances in ART and the increased proportion of medically assisted conceptions in older women have both contributed to the steep increase in the incidence of multiple pregnancies since the 1980s^9,10^. In twin pregnancy, maternal complications including GDM, hypertension, hemorrhage, cesarean delivery, and postpartum depression, as well as perinatal complications including preterm birth, perinatal mortality, and neurodevelopmental impairments, occur more frequently than in singleton pregnancy^11^. Although several countries have seen a reduction in multiple birth rate after setting strategies involving reducing the number of embryos transferred^12^, the multiple birth rate in Korea has exhibited an increasing tendency to date^13^.

There is increasing evidence that non-alcoholic fatty liver disease (NAFLD) in early pregnancy is an independent risk factor for GDM, with a result as patients who developed GDM had high levels of AST, GGT and ALT^14^. Since the liver is an important organ to maintain the glucose homeostasis and insulin resistance, GDM is a strong risk factor to the future NAFLD^15,16^. Obstetric cholestasis and acute fatty liver have a higher risk of occurrence in twin pregnancy than in singleton pregnancy^17^. Our previous study demonstrated that elevated pregravid liver enzyme levels are associated with a risk of developing GDM in singleton pregnancies^18^. However, the association between pregravid liver enzyme levels and the development of GDM in twin pregnancies has to date never been investigated. Thus, this study aimed to investigate the associations between pregravid liver enzyme levels and the risk of GDM + IT or GDM − IT in a subsequent twin pregnancy.

## Material and methods

The methods have been fully described in the previous report^18^. Briefly, women who developed GDM − IT were identified by having more than three claims of GDM (International Classification of Diseases, 10th Revision [ICD-10] codes O24.4 or O24.9), without prescriptions of insulin or oral diabetic medication. Women of GDM + IT were identified by prescription of insulin. Variables in the health interview and health examination, including definitions and classifications of pre-pregnancy factors from the National Health Screening Examination (NHSE) database were also used as same as descriptions in the previous study^18^. The pre-pregnancy levels of GGT, ALT, and AST were dichotomized into high (Q4) and low (Q1–Q3), and those levels were used as binary variables for subgroup analysis. Subgroup analysis was performed based on pre-pregnancy obesity (body mass index [BMI] ≥ 25.0 kg/m^2^) or metabolic syndrome (MetS). This study was approved by the Institutional Review Board of Seoul St. Mary’s Hospital, Catholic University of Korea (KC19ZESI0530) and Korean National Health Insurance Sharing Service (Approval No. NHIS-2021-1-009). Informed consent requirement was waived by the Institutional Review Board of Seoul St. Mary’s Hospital, Catholic University of Korea, because personal identifying information was not accessed. All research was conducted in accordance with relevant guidelines and regulations.

### Study population

**Figure 1:**
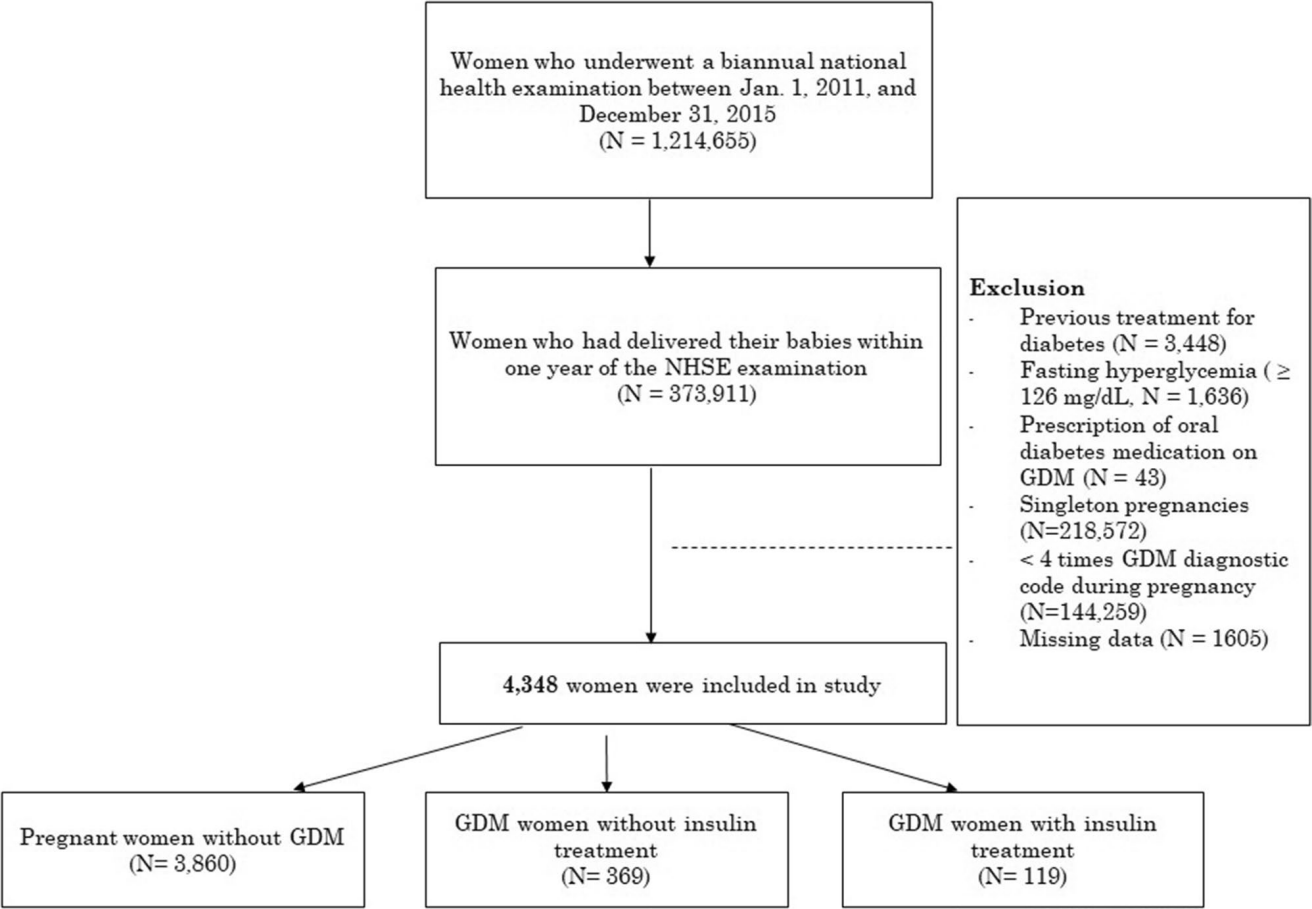
Flowchart of the study population.

Total 373,911 women gave birth within one year of the examination, among 1,214,655 women took the NHSE between January 2011 and December 2015^18^. The final study population consisted of 4348 women who were pregnant with twins, out of 373,911 women (Fig. 1). The other 369,563 women were excluded due to history of treatment for diabetes (N = 3448), fasting hyperglycemia (≥ 126 mg/dL, N = 1636), fewer than four claims with a GDM diagnostic code during pregnancy (N = 144,259), prescription of oral diabetes medication for gestational diabetes (N = 43), missing data concerning BMI or metabolic status (N = 1605), or singleton pregnancies (N = 218,572).

### Statistical analyses

Each variable was checked for normality using the Kolmogorov–Smirnov equality of distributions test. There are expressed as means ± standard deviations or as numbers (percentages). Differences between groups (non-GDM vs. GDM − IT and non-GDM vs. GDM + IT) were analyzed using an independent t-test for continuous variables and the chi-square test for categorical variables. After controlling for covariates of maternal age, parity, smoking, alcohol consumption, income level, exercise status, BMI, and metabolic syndrome, adjusted odds ratios (aOR) and 95% confidence intervals (CI) were estimated for GDM − IT or GDM + IT development, in multivariate logistic regression analysis. In subgroups based on BMI or MetS, multivariate logistic regression analysis was performed to estimate the adjusted ORs for the development of GDM − IT or GDM + IT according to GGT, ALT, or both enzyme levels (high or low). All tests were two-sided, and a *P*-value < 0.05 was considered statistically significant. Statistical analysis was performed using SAS 9.4.

### Ethical approval

This study was approved by the Institutional Review Board of Seoul St. Mary’s Hospital, Catholic University of Korea (KC19ZESI0530) and Korean National Health Insurance Sharing Service (Approval No. NHIS-2021-1-009). Informed consent requirement was waived by the Institutional Review Board of Seoul St. Mary’s Hospital, Catholic University of Korea, because personal identifying information was not accessed. All research was performed in accordance with relevant guidelines and regulations.

## Results

### Characteristics of the study participants

Among the total population of 4348 twin pregnant women, 3860 (88.78%) women in the non-GDM group, 369 (8.49%) in the GDM − IT group were included, and 119 (2.74%) were contained in the GDM + IT group (Table 1). Among the study participants, 10.25% of women had pregravid obesity and 2.67% of women had pregravid MetS. Women in the GDM − IT and GDM + IT groups were significantly older than women in the non-GDM group. When compared to the non-GDM group, the two GDM groups had significantly higher rates of obesity, dyslipidemia, family history of DM, metabolic syndrome, abdominal obesity, hypertriglyceridemia, and low HDL-cholesterol. Mean levels of fasting glucose, ALT, and GGT were significantly higher in the GDM − IT and GDM + IT groups, but the mean level of AST was significantly higher in the GDM + IT group only, and not in the GDM − IT group, when compared to the non-GDM group. There were significant differences in smoking history, income level, and blood pressure between the non-GDM and GDM + IT groups, but not between the non-GDM and GDM − IT groups.

**Table 1:** Characteristics of the study participants.

	Non-GDM n = 3860	GDM − IT n = 369	GDM + IT n = 119	^d^*P*-value	^e^*P*-value
**Age (years)**	31.22 ± 3.65	32.48 ± 3.57	33.04 ± 3.60	< 0.001	< 0.001
≥ 35, n (%)	644 (16.68)	92 (24.93)	35 (29.41)	< 0.001	< 0.001
Nulliparity, n (%)	726 (18.81)	69 (18.7)	26 (21.85)	0.959	0.404
**Smoke**				0.329	< 0.001
Never	3645 (94.43)	343 (92.95)	100 (84.03)		
Past	122 (3.16)	17 (4.61)	9 (7.56)		
Current	93 (2.41)	9 (2.44)	10 (8.4)		
**Drink**				0.686	0.454
Non	2339 (60.6)	232 (62.87)	66 (55.46)		
Mild	1473 (38.16)	133 (36.04)	52 (43.7)		
Heavy	48 (1.24)	4 (1.08)	1 (0.84)		
Regular exercise, n (%)	382 (9.9)	42 (11.38)	16 (13.45)	0.364	0.204
Low income level (< 20%), n (%)	563 (14.59)	61 (16.53)	27 (22.69)	0.314	0.014
**BMI (kg/m**^**2**^**)**	21.18 ± 2.8	21.92 ± 3.43	23.41 ± 4.28	< 0.001	< 0.001
< 18.5, n (%)	519 (13.45)	38 (10.3)	6 (5.04)	< 0.001	< 0.001
18.5–23, n (%)	2531 (65.57)	224 (60.7)	66 (55.46)		
23–25, n (%)	451 (11.68)	53 (14.36)	14 (11.76)		
25–30, n (%)	312 (8.08)	41 (11.11)	22 (18.49)		
≥ 30, n (%)	47 (1.22)	13 (3.52)	11 (9.24)		
Dyslipidemia^b^, n (%)	111 (2.88)	19 (5.15)	11 (9.24)	0.016	< 0.001
Family history of diabetes, n (%)	348 (12.46)	62 (21.45)	24 (28.24)	< 0.001	< 0.001
Metabolic syndrome, n (%)	81 (2.1)	19 (5.15)	16 (13.45)	< 0.001	< 0.001
Abdominal obesity^c^, n (%)	178 (4.61)	30 (8.13)	17 (14.29)	0.003	< 0.001
HDL (< 50 mg/dL), n (%)	678 (17.56)	82 (22.22)	42 (35.29)	0.026	< 0.001
TG (< 150 mg/dL), n (%)	287 (7.44)	41 (11.11)	24 (20.17)	0.012	< 0.001
History of Stroke, n (%)	3 (0.12)	0 (0)	0 (0)	0.575	0.769
History of Heart disease, n (%)	6 (0.23)	0 (0)	0 (0)	0.428	0.678
AST^a^ (U/L)	18.52 (18.35–18.68)	19 (18.45–19.56)	19.69 (18.49–20.98)	0.093	0.019
ALT^a^ (U/L)	13.67 (13.47–13.87)	15.07 (14.33–15.84)	17.33 (15.79–19.02)	< 0.001	< 0.001
GGT^a^ (U/L)	14.59 (14.39–14.8)	15.8 (15.09–16.55)	18.16 (16.62–19.83)	< 0.001	< 0.001
Fasting glucose (mg/dL)	87.61 ± 9.15	90.3 ± 9.99	95.41 ± 12.48	< 0.001	< 0.001
Waist circumference (cm)	70.72 ± 7.39	72.25 ± 8.12	75.65 ± 9.81	< 0.001	< 0.001
Systolic BP (mmHg)	109.97 ± 10.71	110.93 ± 11.05	113.06 ± 13.24	0.103	0.002
Diastolic BP (mmHg)	69.18 ± 8.17	69.56 ± 8.39	72.35 ± 9.27	0.398	< 0.001

Values are expressed as mean ± standard deviation, n (%), or ^a^geometric mean (95% confidence interval).

^b^Dyslipidemia included known treatment for hyperlipidemia.

^c^Abdominal obesity was defined as high waist circumference (≥ 85 cm).

^d^*p*-value: significance probability between non-GDM group and GDM − IT group.

^e^*p*-value: significance probability between non-GDM group and GDM + IT group.

*GDM* gestational diabetes mellitus, *BMI* body mass index, *HDL* high-density lipoprotein, *TG* triglyceride, *AST* aspartate aminotransferase, *ALT* alkaline phosphatase, *GGT* gamma glutamyltransferase, *BP* blood pressure.

*Non-GDM* pregnant women without GDM, *GDM − IT* GDM pregnant women without insulin treatment, *GDM* + *IT* GDM pregnant women with insulin treatment.

### GDM risks in a subsequent twin pregnancy, according to pregravid liver enzyme levels

Pregravid levels of AST, ALT, and GGT across all participants were divided into 4 quartiles. In multivariate logistic regression analysis, women had significantly increased ORs for developing GDM − IT and GDM + IT in twin pregnancy when they were in the 4th quartile for levels of ALT (OR 1.629, 95% CI 1.12–2.368 and OR 2.714, 95% CI 1.308–5.633, respectively) and GGT (OR 1.699, 95% CI 1.18–2.446 and OR 2.126, 95% CI 1.098–4.114, respectively), but not AST (OR 1.065, 95% CI 0.734–1.546 and OR 1.349, 95% CI 0.677–2.691, respectively), compared to women in the lower quartiles of each liver enzyme, after adjusting for family history of DM, dyslipidemia, waist circumference, maternal age, parity, smoking, alcohol consumption, income level, exercise status, BMI, and MetS (Table 2). Pregravid AST level was not associated with the development of GDM − IT or of GDM + IT.

**Table 2:** Adjusted Odds ratios for developing GDM − IT and GDM + IT in women with twin pregnancy according to pregravid liver enzyme levels.

	Non-GDM n = 3860	GDM − IT n = 369	GDM + IT n = 119	OR^a^ (95% CI)	OR^b^ (95% CI)
**AST**					
Q1 (< 16)	630 (22.56)	60 (20.76)	14 (16.47)	1 (Ref.)	1 (Ref.)
Q2 (< 19)	877 (31.4)	84 (29.07)	25 (29.41)	0.977 (0.689, 1.387)	1.194 (0.608, 2.343)
Q3 (< 22)	693 (24.81)	76 (26.3)	20 (23.53)	1.114 (0.777, 1.598)	1.141 (0.558, 2.335)
Q4 (≥ 22)	593 (21.23)	69 (23.88)	26 (30.59)	1.065 (0.734, 1.546)	1.349 (0.677, 2.691)
**ALT**					
Q1 (< 11)	741 (26.53)	49 (16.96)	10 (11.76)	1 (Ref.)	1 (Ref.)
Q2 (< 13)	561 (20.09)	64 (22.15)	15 (17.65)	1.662 (1.125, 2.456)	1.871 (0.828, 4.228)
Q3 (< 17)	781 (27.96)	83 (28.72)	17 (20)	1.434 (0.989, 2.079)	1.278 (0.575, 2.838)
Q4 (≥ 17)	710 (25.42)	93 (32.18)	43 (50.59)	1.629 (1.12, 2.368)	2.714 (1.308, 5.633)
**GGT**					
Q1 (< 12)	804 (28.79)	56 (19.38)	14 (16.47)	1 (Ref.)	1 (Ref.)
Q2 (< 14)	552 (19.76)	54 (18.69)	12 (14.12)	1.431 (0.966, 2.12)	1.193 (0.542, 2.625)
Q3 (< 18)	736 (26.35)	86 (29.76)	17 (20)	1.676 (1.174, 2.391)	1.205 (0.582, 2.494)
Q4 (≥ 18)	701 (25.1)	93 (32.18)	42 (49.41)	1.699 (1.18, 2.446)	2.126 (1.098, 4.114)

Adjusted for maternal age, parity, smoking, alcohol consumption, income level, exercise status, BMI, and metabolic syndrome.

^a^ORs between non-GDM group and GDM − IT group.

^b^ORs between non-GDM group and GDM + IT group.

*ALT* alanine aminotransferase, *AST* aspartate aminotransferase, *GDM* gestational diabetes mellitus, *GGT* gamma-glutamyltransferase, *OR* odds ratio, *CI* Confidence interval, *Q* quartile.

*Non-GDM* pregnant women without GDM, *GDM − IT* GDM pregnant women without insulin treatment, *GDM* + *IT* GDM pregnant women with insulin treatment.

### GDM risks in a subsequent twin pregnancy, according to pregravid liver enzyme levels and obesity

In the subgroup analysis, women were grouped based on obesity status (obese, BMI ≥ 25 kg/m^2^, or not obese, BMI < 25 kg/m^2^) and GGT levels (low, < 18 U/L, or high, ≥ 18 U/L). Women with pregravid obesity and high GGT levels showed importantly elevated ORs for GDM − IT (OR 1.811, 95% CI 1.069–3.07) and GDM + IT (OR 3.011, 95% CI 1.293–7.009) in comparison to women without pregravid obesity and with low GGT levels, in multivariate logistic regression analysis adjusted for maternal age, parity, smoking, alcohol consumption, income level, exercise status, and MetS (Table 3). When women were arranged by pregravid obesity and ALT levels (low, < 17 U/L, or high, ≥ 17 U/L), women with pregravid obesity and high ALT levels also showed significantly raised ORs for GDM − IT (OR 1.747, 95% CI 1.021–2.992), and GDM + IT (OR 3.193, 95% CI 1.381–7.382), as against women without pregravid obesity and with low ALT levels. However, high GGT or ALT in non-obese women was not meaningfully related with an increased risk for GDM − IT or GDM + IT.

**Table 3:** GDM risks in a subsequent twin pregnancy, according to pregravid liver enzyme levels and obesity.

	Pregravid liver enzyme levels	OR (95% CI) for GDM − IT
**Obesity**	**GGT levels**	
No (n = 3816)	GGT < 18 U/L	1 (Ref.)
	GGT ≥ 18 U/L	1.156 (0.851, 1.57)
Yes (n = 413)	GGT < 18 U/L	0.74 (0.364, 1.501)
	GGT ≥ 18 U/L	1.811 (1.069, 3.07)
**Obesity**	**ALT levels**	
No (n = 3816)	ALT < 17 U/L	1 (Ref.)
	ALT ≥ 17 U/L	1.146 (0.849, 1.546)
Yes (n = 413)	ALT < 17 U/L	0.832 (0.43, 1.61)
	ALT ≥ 17 U/L	1.747 (1.021, 2.992)

	Pregravid liver enzyme levels	OR (95% CI) for GDM + IT
**Obesity**	**GGT levels**	
No (n = 3537)	GGT < 18 U/L	1 (Ref.)
	GGT ≥ 18 U/L	1.602 (0.869, 2.955)
Yes (n = 392)	GGT < 18 U/L	1.613 (0.594, 4.382)
	GGT ≥ 18 U/L	3.011 (1.293, 7.009)
**Obesity**	**ALT levels**	
No (n = 3537)	ALT < 17 U/L	1 (Ref.)
	ALT ≥ 17 U/L	1.21 (0.64, 2.288)
Yes (n = 392)	ALT < 17 U/L	1.147 (0.401, 3.283)
	ALT ≥ 17 U/L	3.193 (1.381, 7.382)

Adjusted for maternal age, parity, smoking, alcohol consumption, income level, exercise status, and metabolic syndrome.

*ALT* alanine aminotransferase, *GDM* gestational diabetes mellitus, *GGT* gamma-glutamyltransferase, *OR* odds ratio, *CI* Confidence interval.

*Non-GDM* pregnant women without GDM, *GDM − IT* GDM pregnant women without insulin treatment, *GDM* + *IT* GDM pregnant women with insulin treatment.

### GDM risks in a subsequent twin pregnancy, according to pregravid liver enzyme levels and MetS

In the other subgroup analysis, women were gathered and divied by pregravid MetS and GGT levels (low, < 18 U/L, or high, ≥ 18 U/L) or ALT levels (low, < 17 U/L, or high, ≥ 17 U/L). ORs for GDM + IT in women with pregravid MetS and high GGT levels (OR 5.142, 95% CI 1.878–14.081) were significantly increased, but not for GDM − IT (OR 2.328, 95% CI 0.997–5.438), contrast to women without pregravid MetS and with low GGT levels, in multivariate logistic regression analysis adjusted for maternal age, parity, smoking, alcohol consumption, income level, exercise status, and BMI (Table 4). When classified by pregravid MetS and ALT levels, ORs of GDM + IT in women with MetS, regardless of ALT level, notably raised as compared to women without pregravid MetS and with low ALT. Women with MetS and low ALT had significantly increased ORs for GDM − IT (OR 3.344, 95% CI 1.378–8.115), compared to women without pregravid MetS and with low ALT.

**Table 4:** GDM risks in a subsequent twin pregnancy, according to pregravid liver enzyme levels and metabolic syndrome.

	Pregravid liver enzyme levels	OR (95% CI) for GDM − IT
**Metabolic syndrome**	**GGT levels**	
No (n = 4129)	GGT < 18 U/L	1 (Ref.)
	GGT ≥ 18 U/L	1.3 (0.981, 1.724)
Yes (n = 100)	GGT < 18 U/L	2.528 (0.986, 6.481)
	GGT ≥ 18 U/L	2.328 (0.997, 5.438)
**Metabolic syndrome**	**ALT levels**	
No (n = 4129)	ALT < 17 U/L	1 (Ref.)
	ALT ≥ 17 U/L	1.287 (0.976, 1.697)
Yes (n = 100)	ALT < 17 U/L	3.344 (1.378, 8.115)
	ALT ≥ 17 U/L	1.789 (0.74, 4.329)

	Pregravid liver enzyme levels	OR (95% CI) for GDM + IT
**Metabolic syndrome**	**GGT levels**	
No (n = 3882)	GGT < 18 U/L	1 (Ref.)
	GGT ≥ 18 U/L	1.646 (0.952, 2.848)
Yes (n = 97)	GGT < 18 U/L	2.858 (0.604, 13.53)
	GGT ≥ 18 U/L	5.142 (1.878, 14.081)
**Metabolic syndrome**	**ALT levels**	
No (n = 3882)	ALT < 17 U/L	1 (Ref.)
	ALT ≥ 17 U/L	1.585 (0.917, 2.739)
Yes (n = 97)	ALT < 17 U/L	4.836 (1.233, 18.962)
	ALT ≥ 17 U/L	3.991 (1.45, 10.987)

Adjusted for maternal age, parity, smoking, alcohol consumption, income level, exercise status, and BMI.

*ALT* alanine aminotransferase, *GDM* gestational diabetes mellitus, *GGT* gamma-glutamyltransferase, *OR* odds ratio, *CI* Confidence interval.

*Non-GDM* pregnant women without GDM, *GDM − IT* GDM pregnant women without insulin treatment, *GDM* + *IT* GDM pregnant women with insulin treatment.

### GDM risks in a subsequent twin pregnancy, according to pregravid liver enzyme levels, obesity and MetS

**Figure 2:**
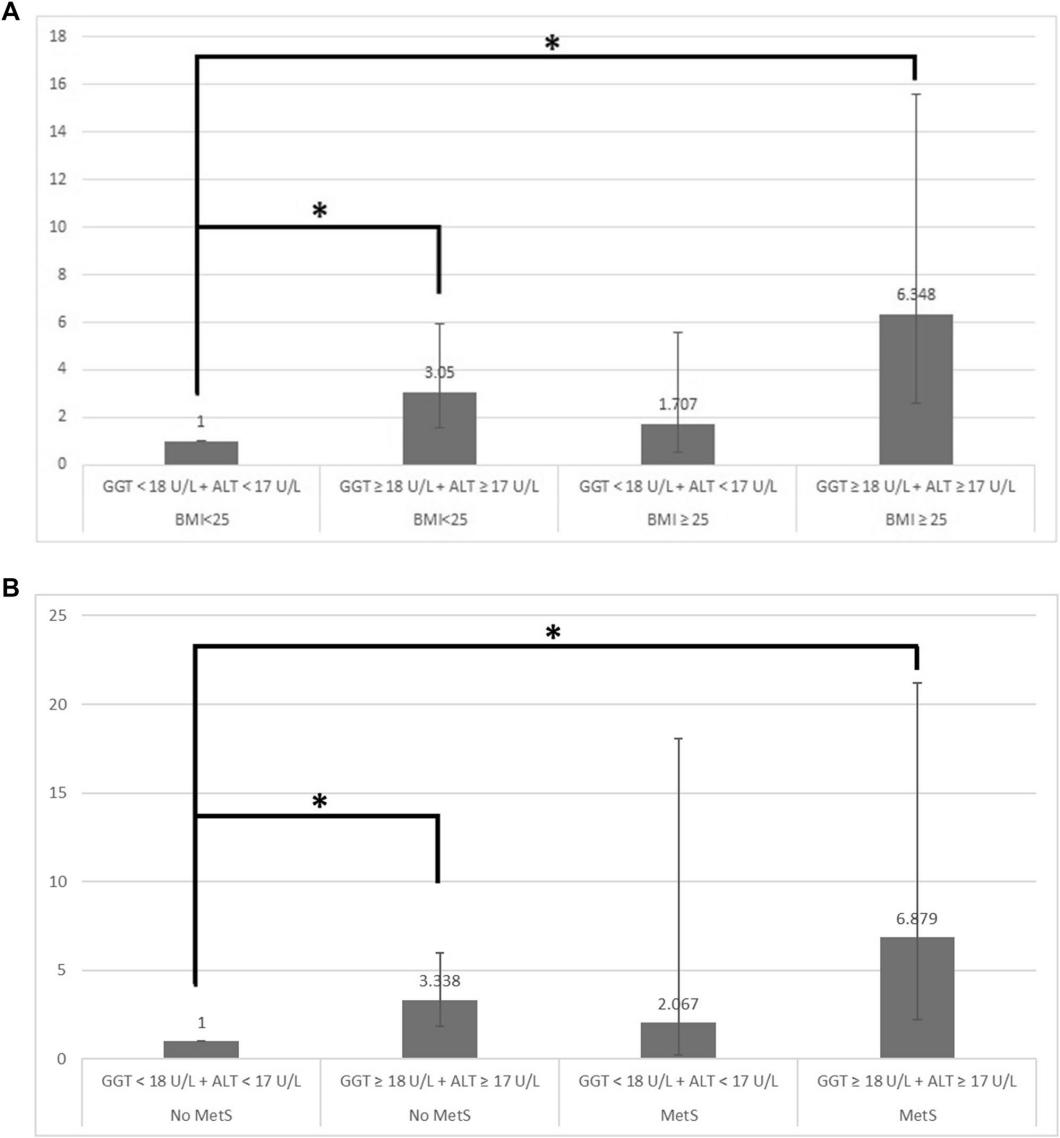
Odd ratios for developing GDM + IT in women with twin pregnancy, according to (**A**) pregravid obesity and liver enzyme levels, and (**B**) pregravid metabolic syndrome and liver enzyme levels.

Women with high levels of GGT and ALT had increased ORs for the development of GDM + IT not only in obese women (OR 6.348, 95% CI 2.579–15.625), but also in non-obese women (OR 3.05, 95% CI 1.565–5.946) when compared to women without obesity and with low enzyme levels (Fig. 2 and Supplementary 1). ORs for the development of GDM + IT in women with high levels of GGT and ALT were higher than women without MetS and with low levels of GGT and ALT, whether they were with MetS (OR 6.879, 95% CI 2.232–21.204) or without MetS (OR 3.338, 95% CI 1.86–5.992). However, there were no significant differences in the risks for development of GDM − IT, irrespective of pregravid obesity or MetS and enzyme levels of ALT and GGT (Table 5).

**Table 5:** GDM − IT risks in a subsequent twin pregnancy, according to pregravid liver enzyme levels, obesity and metabolic syndrome.

Pregravid liver enzyme levels		OR (95% CI) for GDM − IT
**Obesity**		
No (n = 3816)	GGT < 18 U/L + ALT < 17 U/L	1 (Ref.)
	GGT ≥ 18 U/L + ALT ≥ 17 U/L	1.161^a^ (0.7, 1.926)
Yes (n = 413)	GGT < 18 U/L + ALT < 17 U/L	0.388^a^ (0.115, 1.309)
	GGT ≥ 18 U/L + ALT ≥ 17 U/L	1.427^a^ (0.679, 2.997)
**Metabolic syndrome**		
No (n = 4129)	GGT < 18 U/L + ALT < 17 U/L	1 (Ref.)
	GGT ≥ 18 U/L + ALT ≥ 17 U/L	1.37^b^ (0.875, 2.145)
Yes (n = 100)	GGT < 18 U/L + ALT < 17 U/L	2.452^b^ (0.675, 8.914)
	GGT ≥ 18 U/L + ALT ≥ 17 U/L	1.689^b^ (0.581, 4.913)

^a^Adjusted for maternal age, parity, smoking, alcohol consumption, income level, exercise status, and metabolic syndrome.

^b^Adjusted for maternal age, parity, smoking, alcohol consumption, income level, exercise status, and BMI.

*ALT* alanine aminotransferase, *GDM* gestational diabetes mellitus, *GGT* gamma-glutamyltransferase, *OR* odds ratio, *CI* Confidence interval.

*Non-GDM* pregnant women without GDM, *GDM − IT* GDM pregnant women without insulin treatment.

## Discussion

In this study, we investigated the association between pregravid liver enzyme levels and the risk of GDM + IT or GDM − IT in a subsequent twin pregnancy. Our main findings are: (1) High levels of pregravid GGT (Q4, ≥ 18 U/L) showed a significant association with the development of GDM + IT in women with pregravid obesity or MetS. (2) High levels of pregravid ALT (Q4, ≥ 17 U/L) showed a significant association with the development of GDM + IT in women with pregravid obesity, but not in women with MetS. (3) High levels of pregravid GGT or ALT were associated with the development of GDM − IT in women with pregravid obesity, but not in women with pregravid MetS. (4) High levels of GGT and ALT before pregnancy were associated with GDM + IT, regardless of pregravid obesity or MetS. However, there was no association between high levels of either enzyme before pregnancy and GDM − IT. (5) Pregravid AST level was not associated with GDM − IT or GDM + IT in a subsequent twin pregnancy.

Several studies have suggested that twin pregnancies are associated with an increased risk of GDM compared to singleton pregnancies, due to greater placental mass and higher levels of the diabetogenic hormone, human placental lactogen^11,19^. However, a previous study reported that twin pregnancy is associated with an increased risk of GDM − IT, but not GDM + IT^20^. In this study, incidences of overall GDM, GDM − IT, and GDM + IT in women pregnant with twins were 11.2%, 8.49%, and 2.74%, respectively. In our original study including both singleton and multiple pregnancies, we reported incidences of overall GDM, GDM − IT, and GDM + IT as 6.85%, 5.74%, and 1.11%, respectively^18^. Although we cannot conclude that twin pregnancy is more closely associated with GDM + IT compared to the overall pregnancy population, the high incidence of GDM + IT in twin pregnancies should be recognized because GDM + IT is a strong long-term risk factor of developing T2DM^7^.

Pregravid obesity or MetS are well-known risk factors for GDM^2,21^. However, it was reported that the association between maternal obesity and GDM was weaker in twin pregnancy, compared to the association seen in singleton pregnancy^21,22^. In this present study, we demonstrated that elevated GGT or ALT levels in women with obesity increased the risk of GDM − IT and GDM + IT in subsequent twin pregnancies. In addition, high levels of GGT and ALT were associated with an increased risk of GDM + IT, regardless of pregravid obesity or MetS, in subsequent twin pregnancies.

GDM has been associated with a risk of abnormal glucose metabolism or MetS during early postpartum in both singleton and twin pregnancies^23^. MetS is a known risk factor for the development of T2DM and cardiovascular disease^24^. Several meta-analyses reported that the risk of T2DM after GDM was more than seven-fold, compared to normoglycemic pregnancy^6,25,26^. In addition, insulin treatment in pregnancy was a strong predictor for the development of T2DM^7,27,28^. Therefore, the increased risk for GDM + IT in a subsequent twin pregnancy in women with elevated pregravid levels of GGT, ALT, or both enzymes should be recognized, especially when they had pregravid obesity or MetS.

A prospective study about NAFLD in early pregnancy and the subsequent risk of LGA birthweight demonstrated that steatosis on ultrasound in early pregnancy was related to an increased risk of delivering an LGA infant^28^. The other study also reported that unexplained elevated ALT levels in the first trimester were associated with a fourfold increase in the risk of having a neonate with LGA^29^. While most adverse perinatal outcomes of GDM occur similarly in twin and singleton pregnancies^30^, the risk of LGA in neonates was about twofold higher in twin GDM pregnancies, compared to singleton GDM pregnancies, in a large population study^31^. Neonates with LGA in GDM pregnancies have shown a significantly increased risk of developing MetS^32^ and academic delay in childhood^33^. Previously, incremental increases in ALT within normal range were suggested to be an indirect parameter for hepatic insulin resistance^34^. Several studies have consistently reported that elevated GGT levels during early to middle pregnancy are associated with the development of GDM^35–37^, although associations between ALT and AST and the risk of GDM are still controversial^21,37,38^. Further study may be needed to determine whether women with high pregravid liver enzyme levels a) need a liver ultrasound, b) are at increased risk for delivering an LGA or SGA infant, and c) can decrease their risk of GDM + IT through modification of lifestyle or medication. Many studies have reported that NAFLD has a significant association with MetS^39^. Because ALT and GGT are also associated with the development of T2DM^40,41^, GDM + IT in women with high levels of both enzymes may be associated with a higher risk of T2DM in the future, regardless of obesity or MetS, compared to GDM + IT in women with low levels of ALT and GGT.

This study has several limitations. First, it did not include data about ART or polycystic ovarian syndrome as confounding variables for GDM^4,42–44^, because it could not be evaluated using the available data. The lack of ART data can be the biggest limitation in this study. We used the data from the National Health Insurance claims and NHSE, which does not have information about ART, because ART is not covered by national insurance. However, multiple birth rates in Korea have been increased, mainly from ART, despite of extreme low birth rate in Korea^13^. Although ART has been associated with increased risk of GDM in singleton pregnancies, it is still unclear whether ART increases risk of GDM in multiple pregnancies^4^. It is possible that several medications for ART may have negative influence on liver function in women with elevated pregravid liver enzyme levels. Therefore, prepregnancy health check-up including liver enzyme levels may be required in women who receive ART. In addition, endocrinologists need to consider the risk of GDM + IT in women with elevated pregravid liver enzyme levels, when they counsel the number of transferring embryo and ART. More studies may be needed to investigate association between GDM or GDM + IT and ART in multiple pregnancies, especially in women with elevated liver enzyme levels. Secondly, diagnosis of GDM − IT was based on the ICD-10 codes for GDM because of the deficient data on HbA1c and oral glucose tolerance test results in the nationwide database. To reduce diagnostic faults for GDM − IT, we excluded women with fewer than four claims with GDM. Thirdly, we could not approach information about liver enzyme levels during pregnancy or about pregnancy outcomes such as chorionicity, gestational age at delivery, birth weight, gestational weight gain, and other obstetric complications.

The main strengths of our study are its population-based nature and the large sample size of twin pregnancies. A Swedish national registry study reported that women with pregravid NAFLD had a 2.78-fold risk of GDM in singleton pregnancy^45^. However, the study did not evaluate any association between pregravid NAFLD and GDM + IT, nor in particular, the association in twin pregnancies. This study is the first study to demonstrate the correlation between pregravid liver enzyme levels and GDM + IT in a subsequent twin pregnancy. The second strength is that the study population consisted primarily of Asian women with twin pregnancies. Although diversity regarding interracial marriages has been increasing in Korea, more than 90% of marriages remain between a Korean man and a Korean woman, and about 7% of marriages are between a Korean man and a foreign woman^46^. Moreover, more than 80% of the married immigrant women in Korea are from Asian countries, mostly China, Vietnam, and the Philippines^46^. Women from Asia and those with older age and higher BMI are a known high-risk group for developing T2DM^3,25^. Therefore, identification of a significant risk factor in this high-risk group may be important. Lastly, another strength is that we performed subgroup analyses based on the important confounding variables of pregravid maternal BMI and MetS. Elevated levels of both enzymes, GGT and ALT, prior to pregnancy, were associated with GDM + IT, not only in women with pregravid obesity or MetS, but also in women without pregravid obesity or MetS.

Over recent decades, the prevalence of GDM has increased due to demographic changes in pregnant women such as older age, obesity, and MetS, making the disease an urgent concern worldwide^47^. From a public health point of view, the increased prevalence of GDM could contribute to the growing global health burden of obesity and T2DM. The risk of LGA neonates in GDM pregnancies, more likely in twin GDM pregnancies, may contribute to the bigger burden of obesity, MetS, GDM, and T2DM in the next generation.

## Conclusion

Women with high levels of pregravid GGT and ALT need to recognize their increased risk of GDM+IT, regardless of pregravid obesity or MetS, when they get pregnant twin. Endocrinologists need to consider and explain the risk of GDM+IT in women with elevated pregravid liver enzyme levels, when they counsel the number of transferring embryo and ART. Because GDM+IT is strongly associated with the future risk of T2DM, obstetricians need to monitor weight gain, liver enzyme levels, and development of GDM+IT, during pregnancy and provide education about postpartum weight control and glucose test, in twin pregnant women with high levels of pregravid liver enzymes.

## Supplementary Information

Supplementary Information.

## Abbreviations

ALT: Alanine aminotransferase
AST: Aspartate aminotransferase
BMI: Body mass index
CI: Confidence interval
DM: Diabetes mellitus
GDM: Gestational diabetes mellitus
GDM + IT: Gestational diabetes mellitus with insulin treatment requirement
GDM − IT: Gestational diabetes mellitus without insulin treatment requirement
GGT: Gamma-glutamyltransferase
HDL: High-density lipoprotein
HTN: Hypertension
KNHI: The Korea national health insurance
LDL: Low-density lipoprotein
MetS: Metabolic syndrome
NAFLD: Nonalcoholic fatty liver disease
NHSE: The national health screening examination
OR: Odds ratio
Q: Quartile
TG: Triglycerides
T2DM: Type 2 diabetes mellitus
WC: Waist circumference

## References

[1] Jiwani A, Marseille E, Lohse N, Damm P, Hod M, Kahn JG (2012). Gestational diabetes mellitus: Results from a survey of country prevalence and practices. J. Matern. Fetal Neonatal Med..

[2] Yoo HJ (2016). Influences of body size phenotype on the incidence of gestational diabetes needing prescription; analysis by Korea National Health Insurance (KNHI) claims and the National Health Screening Examination (NHSE) database. Metabolism.

[3] Makgoba M, Savvidou MD, Steer PJ (2012). An analysis of the interrelationship between maternal age, body mass index and racial origin in the development of gestational diabetes mellitus. BJOG.

[4] Wang YA, Nikravan R, Smith HC, Sullivan EA (2013). Higher prevalence of gestational diabetes mellitus following assisted reproduction technology treatment. Hum. Reprod..

[5] Benhalima K, Devlieger R, Van Assche A (2015). Screening and management of gestational diabetes. Best Pract. Res. Clin. Obstet. Gynaecol..

[6] Vounzoulaki E, Khunti K, Abner SC, Tan BK, Davies MJ, Gillies CL (2020). Progression to type 2 diabetes in women with a known history of gestational diabetes: Systematic review and meta-analysis. BMJ.

[7] Lee AJ, Hiscock RJ, Wein P, Walker SP, Permezel M (2007). Gestational diabetes mellitus: Clinical predictors and long-term risk of developing type 2 diabetes: A retrospective cohort study using survival analysis. Diabetes Care.

[8] Cho GJ (2015). Secular trends of gestational diabetes mellitus and changes in its risk factors. PLoS ONE.

[9] Martin JA, Hamilton BE, Osterman MJK (2017). Births in the United States, 2016. NCHS Data Brief.

[10] Black M, Bhattacharya S (2010). Epidemiology of multiple pregnancy and the effect of assisted conception. Semin. Fetal Neonatal Med..

[11] Committee on Practice Bulletins-Obstetrics and Society for Maternal-Fetal Medicine (2016). Practice bulletin No. 169: Multifetal gestations: Twin, triplet, and higher-order multifetal pregnancies. Obstet Gynecol..

[12] Heino A (2016). Variations in multiple birth rates and impact on perinatal outcomes in Europe. PLoS ONE.

[13] Ko HS, Wie JH, Choi SK, Park IY, Park YG, Shin JC (2018). Multiple birth rates of Korea and fetal/neonatal/infant mortality in multiple gestation. PLoS ONE.

[14] Lee SM (2019). Non-alcoholic fatty liver disease in the first trimester and subsequent development of gestational diabetes mellitus. Diabetologia.

[15] Ajmera VH, Gunderson EP, VanWagner LB, Lewis CE, Carr JJ, Terrault NA (2016). Gestational diabetes mellitus is strongly associated with non-alcoholic fatty liver disease. Am. J. Gastroenterol..

[16] Donnelly SR (2019). Prospective study of gestational diabetes and fatty liver scores 9 to 16 years after pregnancy. J. Diabetes.

[17] Rao A, Sairam S, Shehata H (2004). Obstetric complications of twin pregnancies. Best Pract. Res. Clin. Obstet. Gynaecol..

[18] Kim WJ (2020). Influences of pregravid liver enzyme levels on the development of gestational diabetes mellitus. Liver Int..

[19] Almog B, Shehata F, Aljabri S, Levin I, Shalom-Paz E, Shrim A (2011). Placenta weight percentile curves for singleton and twins deliveries. Placenta.

[20] Hiersch L (2018). Incidence and risk factors for gestational diabetes mellitus in twin versus singleton pregnancies. Arch Gynecol. Obstet..

[21] Lucovnik M, Blickstein I, Verdenik I, Trojner-Bregar A, Tul N (2015). Maternal obesity in singleton versus twin gestations: A population-based matched case-control study. J. Matern. Fetal Neonatal Med..

[22] Ram M (2020). The relationship between maternal body mass index and pregnancy outcomes in twin compared with singleton pregnancies. Int. J. Obes. (Lond)..

[23] Guillén-Sacoto MA, Barquiel B, Hillman N, Burgos MA, Herranz L (2019). Metabolic syndrome and impaired glucose metabolism during early postpartum after twin pregnancies complicated by gestational diabetes mellitus: Is the risk comparable to singleton pregnancies?. Diabetes Metab..

[24] Grundy SM (2007). Metabolic syndrome: A multiplex cardiovascular risk factor. J. Clin. Endocrinol. Metab..

[25] Bellamy L, Casas JP, Hingorani AD, Williams D (2009). Type 2 diabetes mellitus after gestational diabetes: A systematic review and meta-analysis. Lancet.

[26] Li Z (2020). Incidence Rate of type 2 diabetes mellitus after gestational diabetes mellitus: A systematic review and meta-analysis of 170,139 women. J. Diabetes Res..

[27] Catalano PM, Vargo KM, Bernstein IM, Amini SB (1991). Incidence and risk factors associated with abnormal postpartum glucose tolerance in women with gestational diabetes. Am. J. Obstet. Gynecol..

[28] Lee SM (2019). Nonalcoholic fatty liver disease is a risk factor for large-for-gestational-age birthweight. PLoS ONE.

[29] Yarrington CD, Cantonwine DE, Seely EW, McElrath TF, Zera CA (2016). The association of early unexplained elevated alanine aminotransferase with large-for-gestational-age birthweight. Am. J. Obstet. Gynecol..

[30] Simões T, Queirós A, Correia L, Rocha T, Dias E, Blickstein I (2011). Gestational diabetes mellitus complicating twin pregnancies. J. Perinat Med..

[31] Hiersch L (2019). Gestational diabetes mellitus is associated with adverse outcomes in twin pregnancies. Am. J. Obstet. Gynecol..

[32] Boney CM, Verma A, Tucker R, Vohr BR (2005). Metabolic syndrome in childhood: association with birth weight, maternal obesity, and gestational diabetes mellitus. Pediatrics.

[33] Duffany KO, McVeigh KH, Lipkind HS, Kershaw TS, Ickovics JR (2020). Large for gestational age and risk for academic delays and learning disabilities: assessing modification by maternal obesity and diabetes. Int. J. Environ. Res. Public Health..

[34] Gómez-Sámano MA, Cuevas-Ramos D, Mehta R, Brau-Figueroa H, Meza-Arana CE, Gulias-Herrero A (2012). Association of alanine aminotransferase levels (ALT) with the hepatic insulin resistance index (HIRI): A cross-sectional study. BMC Endocr. Disord..

[35] Sridhar SB, Xu F, Darbinian J, Quesenberry CP, Ferrara A, Hedderson MM (2014). Pregravid liver enzyme levels and risk of gestational diabetes mellitus during a subsequent pregnancy. Diabetes Care.

[36] Kong M (2018). Higher level of GGT during mid-pregnancy is associated with increased risk of gestational diabetes mellitus. Clin. Endocrinol. (Oxf)..

[37] Zhao W (2020). The association of plasma levels of liver enzymes and risk of gestational diabetes mellitus: A systematic review and dose-response meta-analysis of observational studies. Acta Diabetol..

[38] Leng J (2016). Plasma levels of alanine aminotransferase in the first trimester identify high risk chinese women for gestational diabetes. Sci. Rep..

[39] Rosato V, Masarone M, Dallio M, Federico A, Aglitti A, Persico M (2019). NAFLD and extra-hepatic comorbidities: Current evidence on a multi-organ metabolic syndrome. Int. J. Environ. Res. Public Health..

[40] Lee DH (2003). Gamma-glutamyltransferase is a predictor of incident diabetes and hypertension: the Coronary Artery Risk Development in Young Adults (CARDIA) Study. Clin. Chem..

[41] Sattar N (2004). Elevated alanine aminotransferase predicts new-onset type 2 diabetes independently of classical risk factors, metabolic syndrome, and C-reactive protein in the west of Scotland coronary prevention study. Diabetes.

[42] Celik C, Tasdemir N, Abali R, Bastu E, Yilmaz M (2014). Progression to impaired glucose tolerance or type 2 diabetes mellitus in polycystic ovary syndrome: a controlled follow-up study. Fertil Steril..

[43] Karoli R, Fatima J, Chandra A, Gupta U, Islam FU, Singh G (2013). Prevalence of hepatic steatosis in women with polycystic ovary syndrome. J. Hum. Reprod. Sci..

[44] Bosdou JK (2020). Risk of gestational diabetes mellitus in women achieving singleton pregnancy spontaneously or after ART: A systematic review and meta-analysis. Hum. Reprod. Update.

[45] Hagström H (2016). Adverse outcomes of pregnancy in women with non-alcoholic fatty liver disease. Liver Int..

[46] Ministry of Gender Equality and Family (KR). International marriage status. http://www.index.go.kr/potal/main/EachDtlPageDetail.do?idx_cd=2430. (Accessed 5 Jan 2021).

[47] World Health Organization. Global Report on Diabetes. https://www.who.int/publications/i/item/9789241565257. (Accessed 5 Jan 2021).

