# Supplementary material for: Association between pregravid liver enzyme levels and gestational diabetes in twin pregnancies: a secondary analysis of national cohort study
Source: Sci Rep. 2021 Sep 21;11:18695. doi: 10.1038/s41598-021-98180-9 (PMC8455664; doi:10.1038/s41598-021-98180-9)
Supplement: Supplementary file 1 — Supplementary Information. [file 41598_2021_98180_MOESM1_ESM.docx]

**ORIGINAL ARTICLE**

**Association between Pregravid Liver Enzyme Levels and Gestational Diabetes in Twin Pregnancies: A Secondary Analysis of National Cohort study**

Jae-Young Park^1^, Woo Jeng Kim^1^, Yoo Hyun Chung^2^, Bongseong Kim^3^, Yonggyu Park^3^, In Yang Park^1^, Hyun Sun Ko^1 *^

^1^Department of Obstetrics and Gynecology, Seoul St. Mary’s Hospital, College of Medicine, The Catholic University of Korea, Seoul, Republic of Korea

^2^Department of Obstetrics and Gynecology, Daejeon St. Mary’s Hospital, College of Medicine, The Catholic University of Korea, Seoul, Republic of Korea

^3^Department of Biostatistics, College of Medicine, The Catholic University of Korea, Seoul, Republic of Korea

**Correspondence**

Hyun Sun Ko

Department of Obstetrics and Gynecology, Seoul St. Mary’s Hospital, College of Medicine, The Catholic University of Korea, 222, Banpo-daero, Seocho-gu, Seoul, 06591, Republic of Korea

Tel: +82-2-2258-3021, E-mail: [mongkoko@catholic.ac.kr](mailto:mongkoko@catholic.ac.kr)

**ORCID** Hyun Sun Ko 0000-0001-6310-6206

**[Supp 1.] GDM+IT risks in a subsequent twin pregnancy, according to pregravid liver enzyme levels, obesity and metabolic syndrome**

|  | | **Pregravid liver enzyme levels** | **OR (95% CI) for GDM+IT** |
| --- | --- | --- | --- |
| **Obesity** |  | |  |
| No (n=3537) | GGT < 18 U/L + ALT < 17 U/L | | 1(Ref.) |
|  | GGT ≥ 18 U/L + ALT ≥ 17 U/L | | 3.05 ^a^ (1.565, 5.946) |
| Yes (n=392) | GGT < 18 U/L + ALT < 17 U/L | | 1.707 ^a^ (0.522, 5.579) |
|  | GGT ≥ 18 U/L + ALT ≥ 17 U/L | | 6.348 ^a^ (2.579, 15.625) |
| **Metabolic syndrome** |  | |  |
| No (n=3882) | GGT < 18 U/L + ALT < 17 U/L | | 1(Ref.) |
|  | GGT ≥ 18 U/L + ALT ≥ 17 U/L | | 3.338^b^ (1.86, 5.992) |
| Yes (n=97) | GGT < 18 U/L + ALT < 17 U/L | | 2.067^b^ (0.237, 18.055) |
|  | GGT ≥ 18 U/L + ALT ≥ 17 U/L | | 6.879^b^ (2.232, 21.204) |

^a^Adjusted for maternal age, parity, smoking, alcohol consumption, income level, exercise status, and metabolic syndrome.

^b^Adjusted for maternal age, parity, smoking, alcohol consumption, income level, exercise status, and BMI.

ALT: alanine aminotransferase; GDM: gestational diabetes mellitus; GGT: gamma-glutamyltransferase; OR: odds ratio; CI: Confidence interval.

Non-GDM: pregnant women without GDM; GDM+IT: GDM pregnant women with insulin treatment.
